# Risk Factors for Hepatocellular Carcinoma Recurrence and Survival after Liver Transplantation in Patients with HCV-Related Cirrhosis

**DOI:** 10.1155/2020/1487593

**Published:** 2020-10-17

**Authors:** Raphael Iglesias de Oliveira Vidal, Edison Iglesias de Oliveira Vidal, Basilio de Bragança Pereira, Cachimo Combo Assane, Alexandre Ribeiro, Emilia Matos do Nascimento, Fernando Gomes Romeiro, Joaquim Ribeiro Filho

**Affiliations:** ^1^Department of Surgery, Faculty of Medicine, Federal University of Rio de Janeiro (UFRJ), Rua Rodolpho Paulo Rocco, 255-Cidade Universitária, Ilha do Fundão, Rio de Janeiro, RJ, Brazil 21941-902; ^2^Internal Medicine Department, Botucatu Medical School, Sao Paulo State University (UNESP), Av. Prof. Mario Rubens Guimaraes Montenegro, S/N, Botucatu, SP, Brazil 18618-687; ^3^Preventive Medicine Department, Faculty of Medicine, Federal University of Rio de Janeiro (UFRJ), Cidade Universitária, Ilha do Fundão, P.O. Box: 68507, Rio de Janeiro, RJ, Brazil 21941-972; ^4^Department of Mathematics and Informatics, Faculty of Sciences, Universidade Eduardo Mondlane, Av. Julius Nyerere/Campus 3453, P.O. Box 257, Maputo, Mozambique; ^5^Centro Universitário da Zona Oeste, UEZO-Unidade de Engenharia de Produção, Engenharia de Produção, Avenida Manuel Caldeira de Alvarenga, Campo Grande, Rio de Janeiro, RJ, Brazil 23070-200

## Abstract

**Purpose:**

We aimed to identify prognostic factors for survival and recurrence of hepatocellular carcinoma (HCC) after liver transplantation (LT) for patients with HCC and hepatitis C virus-related cirrhosis (HCV-cirrhosis).

**Methods:**

This retrospective cohort study followed all adult patients with HCV-cirrhosis who underwent LT because of HCC or had incidental HCC identified through pathologic examination of the explanted liver at a university hospital in Rio de Janeiro, Brazil, over 11 years (1998-2008). We used Cox regression models to assess the following risk factors regarding HCC recurrence or death after LT: age, Model for End-stage Liver Disease score, Child-Pugh classification, alpha-fetoprotein (AFP), whether patients had undergone locoregional treatment before transplantation, the number of packed red blood cell units (PRBCU) transfused during surgery, the number and size of HCC lesions in the explanted liver, and the presence of microvascular invasion and necrotic areas within HCC lesions.

**Results:**

Seventy-six patients were followed up for a median (interquartile range (IQR)) of 4.4 (0.7-6.6) years. Thirteen (17%) patients had HCC recurrence during the follow-up period, and 26 (34%) died. The median survival time was 6.6 years (95% CI: 2.4-12.0), and the 5-year survival was 52.5% (95% CI: 42.3-65.0%). The final regression model for overall survival included four variables: age (hazard ratio (HR): 1.02, 95% CI: 0.96-1.08, *P* = 0.603), transplantation waiting time (HR: 1.00, 95% CI: 1.00-1.00, *P* = 0.190), preoperative AFP serum levels (HR: 1.01, 95% CI: 1.00-1.02, *P* = 0.006), and whether >4 PRBCU were transfused during surgery (HR: 1.15, 95% CI: 1.05-1.25, *P* = 0.001). The final cause-specific Cox regression model for HCC recurrence included only microvascular invasion (HR: 14.86, 95% CI: 4.47-49.39, *P* < 0.001).

**Conclusion:**

In this study of LT for HCV-cirrhosis, preoperative AFP levels and the number of PRBCU transfused during surgery were associated with overall survival, whereas microvascular invasion with HCC recurrence.

## 1. Introduction

Hepatocellular carcinoma (HCC) represents the third cancer-related cause of death in the world and has an estimated incidence of roughly 750,000 new cases each year [1, 2]. It is associated with high recurrence rates of up to 80% after surgical resection and with lower survival rates when compared with other cancers. That picture is even worse for patients with HCC associated with hepatitis C virus (HCV) infection [3]. Additionally, more than 90% of patients with HCV and HCC also have liver cirrhosis.

Liver transplantation (LT) is considered the best modality of treatment for HCC because it has the potential to clear both the tumor and the underlying liver cirrhosis [4]. In a landmark article published in 1996, the adoption of the Milan criteria for the selection of adult patients with HCC for LT was associated with an improvement in overall survival rates from about 35% in five years to 75% in four years and recurrence rates below 10% [5]. Importantly, these criteria involve only the following: single tumor ≤ 5 cm, or up to 3 foci of the tumor, each ≤ 3 cm, and no evidence of gross vascular invasion or extrahepatic metastasis [6].

Because of the growing demand for LT, several authors advocated the expansion and refinement of prognostic criteria for the selection of eligible patients with HCC [6–9]. Even when the Milan criteria are strictly applied, in real-life cases of LT, it is not rare to find an explanted liver with tumors whose size or number exceeds the limits established by those criteria [10, 11]. In this regard, other variables besides the number and size of tumors in the liver, such as the presence of microvascular invasion, and levels of alpha-fetoprotein (AFP) have also been associated with increased recurrence of HCC after LT [8, 12–16]. A recent systematic review about HCC recurrence after LT concluded that the quality of the studies on this subject was low and that more longitudinal studies providing external validation of risk prediction models in diverse populations are highly needed [17]. Hence, the present study is aimed at examining prognostic factors for mortality and HCC recurrence after LT in a real-life population of patients with HCV-related HCC in Brazil, using the Least Absolute Shrinkage and Selection Operator (LASSO) method [18] to select the most influential variables for survival regression models.

## 2. Materials and Methods

This was a retrospective cohort study based on the review of medical records of all patients undergoing LT at Clementino Fraga Filho Hospital in Rio de Janeiro, Brazil, between January 1, 1998, and December 31, 2008. Our study was approved by the Research Ethics Review Committee of the Faculty of Medicine of the Federal University of Rio de Janeiro under #147/10.

Inclusion criteria involved patients aged 18 years or older who had HCV-cirrhosis and underwent LT because of HCC or had incidental HCC identified through pathologic examination of the explanted liver. For this study, patients were followed up to June 1, 2012.

Preoperative diagnosis of HCC was performed according to the 2000 guidelines of the European Association for the Study of the Liver (EASL) standards [19] and required coincident findings in at least two different radiological examinations (ultrasonography, computed tomography, or magnetic resonance imaging), showing hepatic nodules > 2 cm with evidence of arterial hypervascularization. Alternatively, the EASL guidelines also allowed the noninvasive diagnosis of HCC to be made by a single imaging finding of a focal lesion > 2 cm with arterial hypervascularization when associated with AFP levels > 400 ng/ml. Additionally, according to the standard for selection of patients for transplantation at the time of the study, only patients passing the Milan criteria were considered eligible for LT. All cases of HCC diagnosed preoperatively were confirmed by pathological examination of the explanted liver.

We extracted the following data from patients' medical records: sex, date when the transplantation occurred, age at the date of LT, Model for End-stage Liver Disease (MELD) score (which ranges from 6 to 40, with higher values indicating more severe liver disease), Child-Pugh classification (A, B, and C, with higher levels meaning more severe liver disease), AFP levels before the transplantation (ng/dl), whether patients had undergone locoregional treatment before transplantation (transarterial chemoembolization, radiofrequency ablation, or surgical resection), and the total number of packed red blood cell (PRBC) units that were transfused during surgery. We also reviewed the reports of the pathological examination of the explanted liver for the number and size of HCC lesions, the presence of microvascular invasion, and necrotic areas within HCC lesions. The number of HCC lesions was categorized in the following four groups: single lesion, 2 to 3 lesions, 4 to 5, and more than 5 lesions. Total tumor size was classified as <5 cm, between 5 and 9 cm, and >9 cm. Cases of multicentric HCC were classified as having total tumor size larger than 9 cm and more than 5 lesions.

Additionally, we extracted data from medical records regarding dates of death and when diagnoses of HCC recurrence were made. All cases for which there was not a record of death in their medical records were contacted by phone to confirm they were alive by June 2012.

### 2.1. Statistical Analysis

We described categorical data as absolute numbers and proportions and continuous data as mean and standard deviation (SD) or median and interquartile ranges (IQR), as appropriate [20]. We used a Cox proportional hazards model to assess risk factors for death after LT [21]. We did not use the Fine and Gray subdistribution hazards method to assess risk factors for HCC recurrence accounting for the competing risk of death because that approach is not considered ideal for such purposes [22, 23]. Instead, we used a cause-specific Cox proportional hazards model to assess risk factors for HCC recurrence, as recommended for studies aiming at assessing risk factors for outcomes for which there are one or more competitive events [24]. For that last model, we only included patients who had survived at least one month after LT, as performed by others [25], because it is unlikely that HCC recurrence would be diagnosed in the first month after transplantation.

We used the LASSO method [18] for the selection of variables for both multivariable models, an approach that is considered superior to stepwise methods and that is particularly useful for research contexts where there are a relatively large number of variables in comparison to the total number of observations.

We assessed the proportional hazards assumption of the Cox proportional hazards models through the examination of Schoenfeld residual plots [21].

Six patients with missing data were excluded from the LASSO analyses and from the Cox models that included any variable with missing data.

We adopted a two-sided alpha level of 0.05 for statistical significance and used the R software version 3.6.2 (R Foundation for Statistical Computing, Vienna, Austria) for all statistical analyses.

## 3. Results

Between 1998 and 2008, 373 patients underwent LT at the study hospital. Out of that total, HCV-cirrhosis with HCC was the primary reason for LT for 53 patients. Additionally, 23 patients undergoing LT due to HCV-cirrhosis had their explanted livers diagnosed with incidental HCC. Hence, 76 patients were included in this study.

Fifty-three (70%) patients were male, and the mean (SD) age overall was 56 (7.1) years. Nineteen (25%) patients were classified as Child-Pugh stage C, and the median (IQR) time from inclusion in the transplantation waitlist to surgery was 533 (332 to 846) days. The median (IQR) and maximum duration of follow-up were 4.4 years (0.7 to 6.6 years) and 12 years, respectively.  Table 1 presents further details regarding the demographic and clinical characteristics of patients.

**Figure 1:**
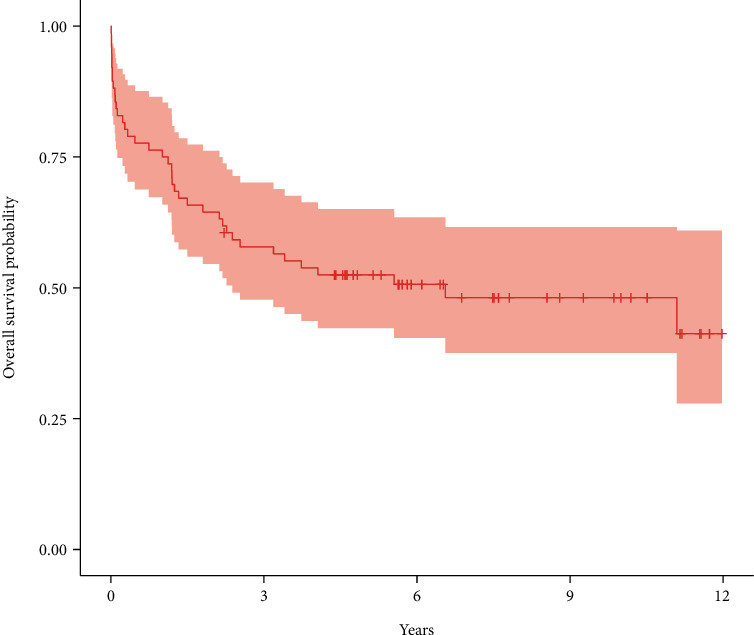
Overall survival with 95% confidence interval.

Thirteen (17%) out of 76 patients had HCC recurrence during the follow-up period, and 26 (34%) died during the timespan of the study. The median survival time was 6.6 years (95% CI: 2.4 to 12.0), and the 5-year survival was 52.5% (95% CI: 42.3% to 65.0%).  Figure 1 shows the Kaplan-Meier overall survival curve after LT. The 5-year cumulative incidence of HCC recurrence was 14.5% (95% CI: 7.7% to 23.5%).

**Figure 2:**
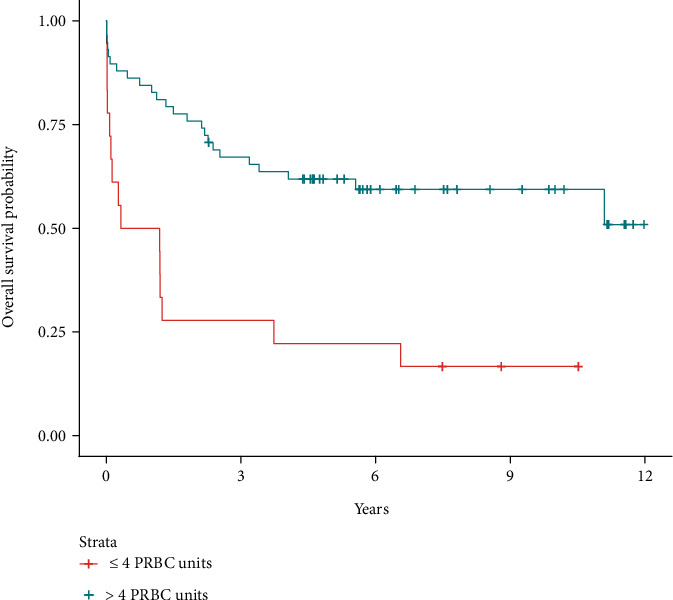
Overall survival according to the number of packed red blood cell units transfused during liver transplantation surgery (HR: 1.15, 95% CI: 1.05 to 1.25, *P* = 0.001).

The results of simple (univariable) Cox regressions for overall survival are shown in Table 2.  Table 3 shows the results of the cause-specific simple Cox regressions for HCC recurrence. The LASSO procedure selected only four variables for the multivariable Cox regression for overall mortality: age, transplantation waiting time, preoperative AFP serum levels, and whether >4 PRBC units were transfused during surgery. The multivariable Cox regression for the overall survival outcome including those four variables (Table 4) showed that intraoperative transfusion of >4 PRBC units was associated with a 15% increased hazard of death (hazard ratio [HR]: 1.15, 95% CI: 1.05 to 1.25, *P* = 0.001) and that every increase of 100 ng/ml of AFP was associated with a 1% increased hazard of death (HR: 1.01, 95% CI: 1.00 to 1.02, *P* = 0.006). Neither age nor transplantation waiting time was significantly associated with the overall survival outcome in the multivariable Cox regression.  Figure 2 depicts the overall survival curves according to the number of PRBC transfused during surgery.

**Figure 3:**
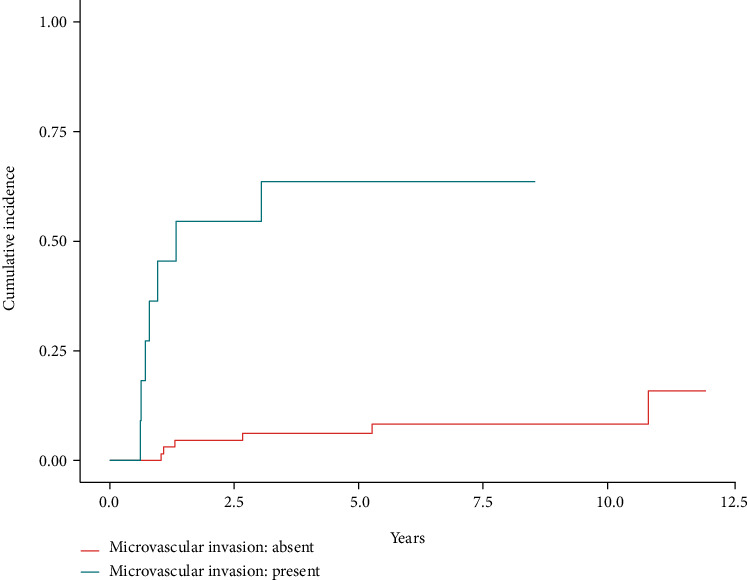
Cumulative incidence of hepatocellular carcinoma recurrence after liver transplantation according to the presence of microvascular invasion in the pathological examination of the explanted liver (HR: 14.86, 95% CI: 4.47 to 49.39, *P* < 0.001).

The LASSO procedure selected only the microvascular invasion variable for the cause-specific Cox regression examining HCC recurrence. The presence of microvascular invasion in the pathological examination of the explanted liver was associated with an increase of almost 15 times in the hazard of HCC recurrence (HR: 14.86, 95% CI: 4.47 to 49.39, *P* < 0.001).  Figure 3 shows the cumulative incidence curves of HCC recurrence according to the presence or absence of microvascular invasion. Schoenfeld residual plots were consistent with the proportional hazards assumption.

## 4. Discussion

The outcomes after LT for patients with liver cirrhosis and HCV depend on variables related not only to the transplantation, such as organ availability, donor selection, allocation strategies, liver disease severity, and local expertise but also on factors associated with HCC survival, such as AFP levels and tumor characteristics. Still, HCV recurrence was a big concern some years ago, when HCV treatment was hardly performed after LT. Therefore, it is vital to analyze the outcomes according to the main underlying liver diseases, such as HCV in the Western world, where HCC accounts for approximately 30% of liver transplants [26].

When LT is proposed for a patient with HCC, one of the main concerns involves the possibility of multicentric recurrence and intrahepatic distant recurrence, which often occur in the first 2 years and are particularly common in HCV-related HCC, contributing to the worse outcomes in this population [3, 26, 27]. Although the need for PRBC transfusion in LT had already been associated with length of hospital stay and acute rejection [28], a recent study found that patients with HCC had a 5 times higher chance of requiring massive intraoperative transfusion of 10 or more PRBC units than patients without HCC [29]. In that study, Danforth et al. [29] evaluated a sample of 124 patients undergoing LT, in whom HCC was the main etiology of liver disease in only 16 (12.9%). Of note, half of our 76 patients with HCV-related HCC required intraoperative transfusions of at least 2 PRBC units and a quarter received 4 or more PRBC. Our results showed that transfusions of more than 4 PRBC units were associated with lower survival in this population, a finding that is consistent with results from previous studies involving other populations of patients undergoing LT [30] and that likely reflects a range of possible factors such as the degree of difficulty of the surgical procedure, the occurrence of intraoperative complications leading to blood loss, and adverse immune effects related to PRBC transfusions [31]. Importantly, our results point towards a possible role of interventions aimed at preventing blood loss and minimizing the need for intraoperative transfusions as a means to improve the outcomes of patients undergoing LT.

AFP is widely recognized as a prognostic predictor of survival for patients with HCC undergoing LT based on studies of patients with heterogeneous underlying causes of liver disease [32]. Our study found that in a population of patients with HCV-cirrhosis, every increase of 100 ng/ml in AFP levels was associated with a 1% increase in the hazard of death and contributes to the literature with information concerning this specific subgroup of patients with HCC.

Rates of HCC recurrence after LT usually vary from 5% to 15% [13, 26, 33]. However, those estimates were derived from heterogeneous samples in hospitals where most patients had an early diagnosis and were submitted to LT with small tumors. For instance, in a long-term study performed by Doyle et al., patients had only a 7% incidence of HCC recurrence, but their sample had different underlying liver diseases and very small tumors (2.3 ± 1.3 cm) [27]. In our study, which was restricted to patients whose HCC was related to HCV-cirrhosis, we found a recurrence rate of 17% after LT, which was lower than the 32.4% recurrence rate described by Bozorgzadeh et al. [34] in their cohort of 37 patients with HCC due to HCV-cirrhosis after a mean follow-up of 37 months after LT.

Although AFP levels, the number of tumor lesions, and their size are considered well-established risk factors for HCC recurrence after LT, we did not find significant associations between those variables and HCC recurrence in our study [35]. The most probable explanation for that lack of association is insufficient statistical power related to our limited sample size. On the other hand, our results showed an almost 15 times higher hazard of HCC recurrence in patients whose pathological examination of their explanted livers revealed microvascular invasion than when that feature was absent. Microvascular invasion was associated with a 3.8- to 4.9-fold increase in HCC recurrence in prior studies with heterogeneous samples [26]. Our results showed a higher impact of microvascular invasion in terms of risk of HCC recurrence for patients whose underlying liver disease was HCV-cirrhosis, which could possibly be explained by particularities of the molecular mechanisms driving hepatocarcinogenesis in HCV-cirrhosis, such as the methylation of multiple genes and the compromise of the DNA damage response [36–40].

Unfortunately, current practice for the diagnosis of microvascular invasion still relies solely on the pathological examination of surgical specimens. However, recent advances in the field of radiology, radiomics, and radiogenomics have shown promising results concerning the noninvasive diagnosis of microvascular invasion [41–44].

Our study has some potential limitations worth noting. First, our sample was relatively small and our analyses may not have had enough statistical power to detect other predictive variables for overall survival and HCC recurrence. Second, our study was restricted to a single center and our findings may not be generalizable to other settings. Third, we were not able to include in our models several variables related to the histopathological and immunohistochemical profile of the HCC. Nevertheless, our study is valuable for providing data on a subgroup of HCC with a single underlying liver disease in the context of a middle-income country, for which little information is available in the medical literature.

## 5. Conclusion

In summary, our results provide evidence of a significant mortality and cancer recurrence burden in a population of patients with HCC associated with HCV-cirrhosis that underwent LT. For that population, the number of PRBC units transfused during surgery and the preoperative AFP serum levels were associated with decreased overall survival, whereas the presence of tumor microvascular invasion was the single most important predictor of HCC recurrence.

## Figures and Tables

**Table 1:** Clinical and demographic characteristics of the study subjects.

	*N* (%)
Sex	
Female	23 (30.3%)
Male	53 (69.7%)
Age	
Mean (SD)	56 (7.1)
Child-Pugh stage	
A	28 (36.8%)
B	29 (38.2%)
C	19 (25.0%)
MELD	
Median (IQR)	14 (9.8 to 16)
Transplantation waiting time (days)	
Median (IQR)	533 (332 to 846)
Missing data	5 (6.6%)
Locoregional therapy before transplantation^∗^	47 (61.8%)
Transarterial chemoembolization	37 (48.7%)
Radio frequency ablation	5 (6.6%)
Liver resection	5 (6.6%)
Number of red blood cell units transfused during transplantation	
Median (IQR)	2.0 (1.0 to 4.0)
Alpha-fetoprotein (ng/ml)	
Median (IQR)	17.2 (5.6 to 67)
Missing data	3 (3.9%)
Number of HCC tumors	
Single tumor	23 (30.3%)
2-3 tumors	19 (25.0%)
4-5 tumors	7 (9.2%)
>5 tumors	27 (35.5%)
Total tumor size	
<5 cm	37 (48.7%)
5-9 cm	10 (13.2%)
>9 cm	29 (38.2%)
Presence of tumor necrosis	
Yes	37 (48.7%)
Missing data	1 (1.3%)
Microvascular invasion	
Yes	11 (14.5%)

SD: standard deviation; IQR: interquartile range; MELD: Model for End-stage Liver Disease; HCC: hepatocellular carcinoma.

**Table 2:** Results of univariable Cox regressions for overall survival.

	HR	95% CI	*P* value
Age	1.02	0.97-1.08	0.378
Sex			
Female	—	—	—
Male	0.68	0.31-1.50	0.336
Transplantation waiting time	1.00	1.00-1.00	0.483
Child-Pugh classification			
Child-Pugh stage A	—	—	—
Child-Pugh stage B	1.27	0.55-2.95	0.572
Child-Pugh stage C	0.50	0.16-1.59	0.240
MELD score	1.00	0.92-1.09	0.990
Alpha-fetoprotein (ng/ml) × 100^a^	1.01	1.00-1.02	0.018
Previous locoregional treatment for HCC	1.55	0.67-3.57	0.307
Number of HCC lesions			
Single tumor	—	—	—
2-3 tumors	1.59	0.58-4.40	0.370
4-5 tumors	1.54	0.40-5.99	0.530
>5 tumors	1.05	0.38-2.89	0.931
Total tumor size			
<5 cm	—	—	—
5-9 cm	0.88	0.25-3.10	0.846
>9 cm	1.05	0.46-2.39	0.913
Incidental HCC diagnosed postoperatively	0.59	0.24-1.48	0.265
Microvascular invasion	0.53	0.13-2.27	0.395
Presence of tumor necrosis	1.44	0.66-3.15	0.355
>4 red blood cells units transfused during transplantation	4.06	1.86-8.86	<0.001

^a^The results reported here for alpha-fetoprotein levels correspond to increases of 100 units of alpha-fetoprotein levels; i.e., the hazard ratio of 1.01 means that every increase of 100 ng/ml is associated with a 1% increase in the hazard of death. HCC: hepatocellular carcinoma; MELD: model for end-stage liver disease.

**Table 3:** Results of simple cause-specific Cox regressions for hepatocellular carcinoma recurrence.

	HR	95% CI	*P* value
Age	0.96	0.89-1.05	0.403
Sex			
Female			
Male	3.71	0.47-28.97	0.212
Transplantation waiting time	1.00	1.00-1.00	0.573
Child-Pugh classification			
Child-Pugh stage A			
Child-Pugh stage B	2.41	0.62-9.32	0.202
Child-Pugh stage C	0.50	0.05-4.81	0.549
MELD score	0.92	0.80-1.06	0.262
Alpha-fetoprotein (ng/ml) × 100^∗^	1.00	0.99-1.01	0.746
Received previous locoregional treatment for HCC	1.28	0.37-4.37	0.695
Number of HCC lesions			
Single tumor			
2-3 tumors	2.58	0.23-28.49	0.439
4-5 tumors	9.05	0.81-100.45	0.073
>5 tumors	5.81	0.70-48.30	0.103
Total tumor size			
<5 cm			
5-9 cm	3.76	0.62-22.64	0.149
>9 cm	3.04	0.76-12.15	0.116
Incidental HCC diagnosed postoperatively	1.13	0.33-3.87	0.842
Microvascular invasion	14.86	4.47-49.39	<0.001
Presence of tumor necrosis	1.44	0.41-5.12	0.569
>4 red blood cells units transfused during transplantation	1.30	0.35-4.92	0.695

^a^The results reported for alpha-fetoprotein levels correspond to increases of 100 units of alpha-fetoprotein levels; i.e., the hazard ratio of 1.01 means that every increase of 100 ng/ml is associated with a 1% increase in the hazard of hepatocellular carcinoma recurrence. HCC: hepatocellular carcinoma; MELD: model for end-stage liver disease.

**Table 4:** Results of multivariable Cox regressions for overall survival.

	HR	95% CI	*P* value
Age	1.02	0.96-1.08	0.603
Transplantation waiting time	1.00	1.00-1.00	0.190
Alpha-fetoprotein (ng/ml) × 100^a^	1.01	1.00-1.02	0.006
>4 red blood cells units transfused during transplantation	1.15	1.05-1.25	0.001

^a^The results reported here for alpha-fetoprotein levels correspond to increases of 100 units of alpha-fetoprotein levels; i.e., the hazard ratio of 1.01 means that every increase of 100 ng/ml is associated with a 1% increase in the hazard of hepatocellular carcinoma recurrence.

## Data Availability

The anonymized dataset is available from the corresponding author upon reasonable request.
